# Stimulus-Selective Regulation of Human Mast Cell Gene Expression, Degranulation and Leukotriene Production by Fluticasone and Salmeterol

**DOI:** 10.1371/journal.pone.0096891

**Published:** 2014-05-12

**Authors:** Adriana Catalli, Victor Karpov, Levente E. Erdos, Brian P. Tancowny, Robert P. Schleimer, Marianna Kulka

**Affiliations:** 1 National Institute for Nanotechnology, National Research Council, Edmonton, Alberta, Canada; 2 Department of Medical Microbiology and Immunology, University of Alberta, Edmonton, Alberta, Canada; 3 Northwestern University Feinberg School of Medicine, Allergy-Immunology Division, Chicago, Ilinois, United States of America; University ofTennessee Health Science Center, United States of America

## Abstract

Despite the fact that glucocorticoids and long acting beta agonists are effective treatments for asthma, their effects on human mast cells (MC) appear to be modest. Although MC are one of the major effector cells in the underlying inflammatory reactions associated with asthma, their regulation by these drugs is not yet fully understood and, in some cases, controversial. Using a human immortalized MC line (LAD2), we studied the effects of fluticasone propionate (FP) and salmeterol (SM), on the release of early and late phase mediators. LAD2 cells were pretreated with FP (100 nM), SM (1 µM), alone and in combination, at various incubation times and subsequently stimulated with agonists substance P, C3a and IgE/anti-IgE. Degranulation was measured by the release of β-hexosaminidase. Cytokine and chemokine expression were measured using quantitative PCR, ELISA and cytometric bead array (CBA) assays. The combination of FP and SM synergistically inhibited degranulation of MC stimulated with substance P (33% inhibition compared to control, n = 3, P<.05). Degranulation was inhibited by FP alone, but not SM, when MC were stimulated with C3a (48% inhibition, n = 3, P<.05). As previously reported, FP and SM did not inhibit degranulation when MC were stimulated with IgE/anti-IgE. FP and SM in combination inhibited substance P-induced release of tumor necrosis factor (TNF), CCL2, and CXCL8 (98%, 99% and 92% inhibition, respectively, n = 4, P<.05). Fluticasone and salmeterol synergistically inhibited mediator production by human MC stimulated with the neuropeptide substance P. This synergistic effect on mast cell signaling may be relevant to the therapeutic benefit of combination therapy in asthma.

## Introduction

Treatment of inflammation relies heavily on the use of glucocorticosteroids, which are presently the most effective drugs available for the management of many severe inflammatory diseases including asthma, rhinitis and chronic obstructive pulmonary disease (COPD), to name just a few [1]–[4]. Many advances have been made in the understanding of the mechanisms of glucocorticoid action; these drugs inhibit the recruitment and activation of inflammatory cells responsible for tissue damage and also inhibit the blood vessel leakage that leads to edema. The advent of topical preparations of glucocorticoids has substantially improved the therapeutic index of these drugs. Glucocorticoids and adrenergic hormones (epinephrine and norepinephrine) interact at both cellular and molecular levels to enhance each other's actions during stress responses. This fact has been exploited in the development of drug preparations combining a glucocorticoid and a long acting beta adrenergic agonist (LABA) for the treatment of asthma [5]–[7].

Early studies on human mast cells by our group have demonstrated that the release of histamine and leukotrienes is not affected by exposure to glucocorticoids [8]–[10]. Thus, the profound ability of glucocorticoids to inhibit allergic late phase responses (LPR) is not likely to be due to the inhibition of mast cell degranulation. The immediate wheal and flare response to allergic skin testing is not inhibited by glucocorticoids. Yet, glucocorticoids inhibit the expression of cytokines by IgE/antigen-activated human mast cells and this may contribute to the ability of glucocorticoids to inhibit LPR [11], [12]. However, there is some evidence that mast cells may respond differently to glucocorticoids when they are activated via non IgE/FcεRI-medated pathways. For example, prednisolone inhibits substance P (SP)-induced histamine release from mouse peritoneal mast cells [13]. Additional information is required regarding the effects of glucocorticoids on the release of inflammatory cytokines and chemokines by mast cells, especially given that cytokine production by mast cells is of great relevance to inflammatory disease.

In contrast to glucocorticoids, it has been known for decades that beta-adrenergic drugs inhibit mast cell degranulation [14]–[16]. Elevation of cAMP inhibits human mast cell activation and may contribute to some of the effects of beta agonists on both bronchoconstriction and airway edema. High concentrations of these drugs are required to inhibit cytokine generation by basophils, however (unpublished observations).

The interactions of glucocorticoids and beta agonists in the regulation of human mast cell function have not been well characterized. In previous studies, we have found that glucocorticoids can influence β-adrenoceptor desensitization on human mast cells [17], [18]. At the very least, we expected that these drugs would be complementary, i.e. glucocorticoids would inhibit cytokine expression and beta agonists would inhibit degranulation. In this case, the combination would be expected to inhibit mast cell inflammatory responses significantly better than either drug alone. Recent studies indicate that beta adrenergic agonists can prime the glucocorticoid receptor and enhance glucocorticoid signaling [19], [20]. In addition, it has been known for decades that glucocorticoids can potentiate adrenergic signaling by a combination of inducing the β2 adrenergic receptor, inhibiting adrenergic desensitization and via other mechanisms [1], [21]. Given the widespread use of combinations of glucocorticoids and LABA, and in recognition of the central importance of mast cells in inflammatory diseases of the airways such as asthma and COPD, it is important to better characterize the interactions of glucocorticoids and LABA as modifiers of mast cell function.

## Materials and Methods

### Human MC culture

LAD2 MC [22] were cultured in serum free media (StemPro-34 SFM, Life Technologies) supplemented with 2 mM L-glutamine, 100 U/ml penicillin, 50 µg/ml streptomycin and 100 ng/ml stem cell factor (SCF). The cell suspensions were seeded at a density of 10^5^ cells/ml and maintained at 37°C and 5% CO_2_. Cells were fed by hemi-depletion of medium once per week.

Human peripheral blood derived CD34+ cells (StemCell Technologies, Vancouver, Canada) were cultured in StemPro-34 SFM supplemented with 2 mM L-glutamine, 50 µg/ml streptomycin, 100 U/ml penicillin, 100 ng/ml SCF, and 100 ng/ml recombinant human IL-6 (PeproTech, Inc., Rocky Hill, NJ). Recombinant human IL-3 (30 ng/ml) was added for the first week. Half of the culture medium was replaced every 7 days. Cultures at 8 to 10 weeks consisted of greater than 99% huMC [23]. Unless otherwise stated, experiments were performed in StemPro-34 SFM complete with 100 ng/mL SCF.

### Treatments

In most cases, unless otherwise stated, LAD2 human mast cells were treated with fluticasone (0.1 nM) or salmeterol (10 nM) or both for 20 hr, then stimulated with SP (1 µg/mL) or C3a (100 ng/mL) for 30 min and β-hexosaminidase release was measured (according to methods described below). For FcεRI-mediated stimulation, LAD2 human mast cells were sensitized with human myeloma IgE (0.5 µg/mL) for 16 hr, then treated with fluticasone (0.1 µM) or salmeterol (1 µM) or both for 20 hr and stimulated with anti-IgE (100 ug/mL) for 30 min and β-hexosaminidase release was measured. In some experiments, LAD2 human mast cells were treated with 0.1, 1, 10 and 100 nM fluticasone and/or salmeterol for 20 hr, then stimulated with SP (0.5 µg/mL) for 30 min and β-hexosaminidase release was measured.

### Degranulation assay

Cells were sensitized overnight with 0.5 µg/mL of human myeloma IgE (Calbiochem). Cells were washed, resuspended in buffer and then stimulated with rabbit anti-IgE (Dako, Carpinteria, CA) or other agonists and incubated at 37°C for 0.5 hr. The β-hexosaminidase released into the supernatants and in cell lysates was quantified by hydrolysis of p-nitrophenyl N-acetyl-β-D-glucosamide (Sigma-Aldrich, St Louis, MO) in 0.1 M sodium citrate buffer (pH 4.5) for 90 min at 37°C. The percentage of β-hexosaminidase release was calculated as a percent of total content. Agonists tested were IgE/anti-IgE, SP (Sigma) and C3a (Calbiochem).

### Real time PCR analysis

Total RNA was isolated from each preparation using the RNeasy Mini Kit (Qiagen Inc. Valencia, CA). Five micrograms of total cellular RNA was reverse transcribed using the Taqman Reverse Transcription reagents and oligoDT primer (Invitrogen). Gene expression was analyzed using real-time PCR on an ABI StepOnePlus system. Fifty ng of cDNA was used in each quantitative PCR assay. Primer sets for PCR amplifications were designed using the Primer Express software (Applied Biosystems). All reactions were performed in triplicate for 40 cycles as per the manufacturer's recommendation. Results are expressed as relative mRNA corrected with reference to GAPDH mRNA as an internal control [24]. Sequences of primers and probes are shown in Table 1.

**Table 1:** qPCR primer/probe sequences.

Gene	Forward primer	Reverse primer	Probe: FAM.TAMRA (GAPDH: MAX/BHQ)
TNF	TCT GGC CCA GGC AGT CA	GCT TGA GGG TTT GCT ACA ACA TC	CTT CTC GAA CCC CGA GTG ACA AGC
IL-8/CXCL8	CTG GCC GTG GCT CTC TTG	TTG GCA AAA CTG TTT AGC ACT CC	CAG CCT TCC TGA TTT CTG CAG CTC TGT GT
CCL1/I-309	GAT TTC TTT CCA TTG TGG GCT CT	CAG GGC AGA AGG AAT GGT GTA	ACA TGG CTT CAC CTG TCC CCG AAA CT
MCP-1/CCL2	TCT CTG CCG CCC TTC TGT	GCC TCT GCA CTG AGA TCT TCC T	CTG CTC ATA GCA GCC ACC TTC ATT CCC
MIP-1β/CCL4	CAG CGC TCT CAG CAC CAA	TTC CTC GCG GTG TAA GAA AAG	CTC AGA CCC TCC CAC CGC CTG C
C3aR	CAG CGG ACT TCA AAA ACT GTC A	GGT GAT GCT GAT GTC AAT AGT CTG T	AGA ATC AAT CCA GCG GTT CTC AAA CGG T
TACR1	CCT CCA TGG CTG CAT TCA AT	CAT TCG TTG TGG ACA GCA TAG G	CAG TGG TGA ACT TC
TACR2	GGC CGT GCC TTG TGC TAC T	ATG CTG ACA AAC ATG GCT GTG A	CAG AAC CTC TTC CCC
TACR3	GTG GCA GTG GCA GTT TTG G	CGC TTG TGG GCC AGG AT	AAATCT CAT CGT CAT CTG GAT
GAPDH	GAT TCC ACC CAT GGC AAA TTC	GGG ATT TCC ATT GAT GAC AAG C	CGT TCT CAG CCT TGA CGG TGC CA

### Flow cytometry

Cells were washed with phosphate buffered saline (PBS), resuspended at 1×10^6^ cells/mL in PBS/0.1% bovine serum albumin (BSA) and incubated with anti-C3aR-PE (BD Bioscience), anti-TACR1, anti-TACR2, or anti-TACR3 (all from Abcam, Cambridge, MA), or appropriate isotype control antibody (BD Biosciences) for 30 min at 4°C. Cells were washed twice and in the cases where the primary antibody was unconjugated (all TACR2 antibodies), anti-rabbit-PE (BD Biosciences) or anti-mouse-PE (BD Biosciences) was added for 30 min at 4°C. Cells were washed twice, resuspended in PBS/0.1% BSA and analyzed on a FACSArray (BD Biosciences).

### Statistical analysis

Each experiment was performed at least 3 separate times, in quadruplicate and values displayed represent mean +/− standard error of the mean. P values were determined by Student's t test (between groups) or one-way ANOVA (comparing more than two groups).

## Results

### FP and SM inhibited human mast cell degranulation

**Figure 1:**
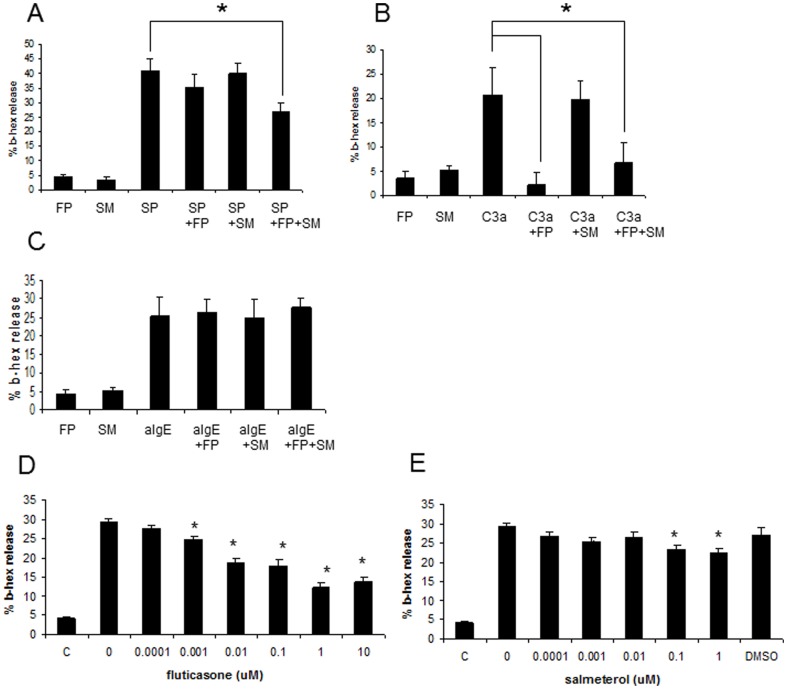
Fluticasone and salmeterol inhibit human mast cell degranulation. **A**, LAD2 human mast cells were treated with fluticasone (0.1 nM) or salmeterol (10 nM) or both for 20 hr, then stimulated with SP (1 ug/mL) for 30 min and β-hexosaminidase release was measured (n = 5, * = p<0.01). **B**, LAD2 human mast cells were treated with fluticasone (0.1 uM) or salmeterol (1 uM) or both for 20 hr, then stimulated with C3a (100 ng/mL) for 30 min and β-hexosaminidase release was measured (n = 5, * = p-value<0.01) **C**, LAD2 human mast cells were sensitized with human myeloma IgE (0.5 ug/mL) for 16 hr, then treated with fluticasone (0.1 uM) or salmeterol (1 uM) or both for 20 hr and stimulated with anti-IgE (100 ug/mL) for 30 min and β-hexosaminidase release was measured (n = 5, * = p-value<0.01). **D**, LAD2 human mast cells were treated with fluticasone for 20 hr, then stimulated with SP (1 ug/mL) for 30 min and β-hexosaminidase release was measured (n = 5, * = p<0.05 when compared to untreated control (C)). **E**, LAD2 human mast cells were treated with salmeterol for 20 hr and stimulated with SP (100 ng/mL) for 30 min and β-hexosaminidase release was measured (n = 5, * = p-value<0.05 when compared to untreated control (C)).

To determine whether FP and SM inhibit human mast cell degranulation, LAD2 human mast cells were pretreated for 20 hr with FP (0.1 nM) and/or SM (10 nM), stimulated with SP, C3a or IgE/anti-IgE and the amount of degranulation was determined by measurement of β-hexosaminidase release (Fig. 1). FP and SM individually had no significant effect on SP-mediated activation of LAD2 degranulation at the concentrations tested (Fig. 1A). However, SM and FP in combination inhibited SP-activated degranulation by approximately 35% (Fig. 1A). FP alone inhibited C3a-activated degranulation by almost 90% whereas SM alone had no effect on C3a-activated degranulation (Fig. 1B). SM and FP in combination inhibited C3a-activated degranulation by 60%. As expected based on published studies, FP and SM individually, or in combination, did not inhibit IgE/anti-IgE-mediated LAD2 degranulation (Fig. 1C).

### SM and FP effects on SP-mediated human mast cell degranulation were concentration-dependent

Since SM and FP synergistically inhibited SP-mediated LAD2 degranulation (Fig. 1A), we next determined the concentration that inhibited degranulation by 50% (IC_50_). At concentrations above 0.1 nM, FP inhibited SP-activated mast cell degranulation in a concentration-dependent manner and had an IC_50_ of approximately 50 nM (Fig. 1D). SM inhibited SP-activated mast cell degranulation at concentrations above 0.01 µM but the effect of SM was not significantly increased at 1 µM and the IC_50_ was not reached (Fig. 1E).

### FP and SM synergistically inhibited SP-activated human mast cell degranulation

**Figure 2:**
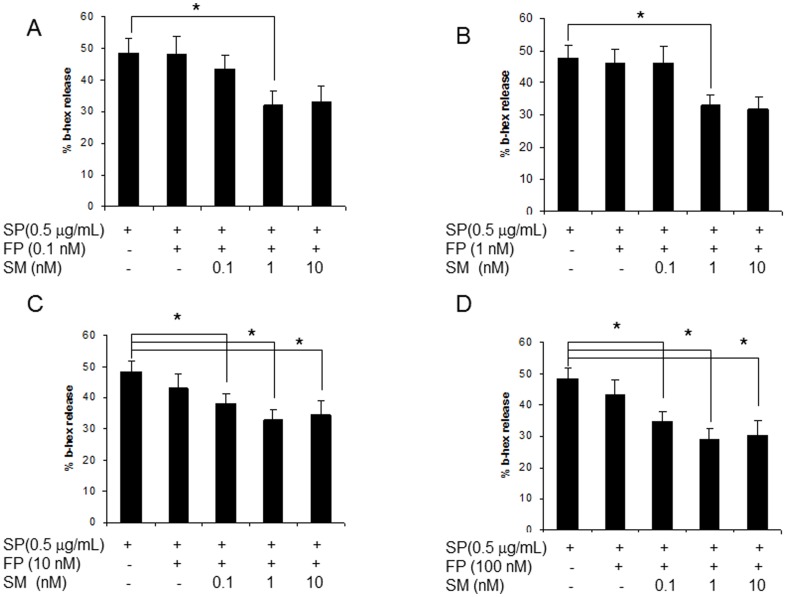
Fluticasone and salmeterol synergistically inhibit mast cell degranulation. **A**, LAD2 human mast cells were treated with 0.1, 1, 10 and 100 nM fluticasone (**A**, **B**, **C** and **D** respectively) and/or salmeterol for 20 hr, then stimulated with SP (0.5 ug/mL) for 30 min and β-hexosaminidase release was measured (n = 5, * = p<0.01).

To determine the optimal concentrations at which FP and SM inhibit SP-induced degranulation, a two-way concentration response study was conducted in which LAD2 cells were pretreated with FP and SM in combination at increasing concentrations. At low concentrations of FP (0.1 and 1 nM), 1 nM SM was required to inhibit degranulation (Fig. 2A and B). However, at higher concentrations of FP (10 and 100 nM), the addition of as little as 0.1 nM SM inhibited degranulation (Fig. 2C and D). Therefore, 1 nM SM enhanced the effect of FP at 0.1 and 1 nM whereas 0.1 nM SM enhanced the effect of FP at 10 and 100 nM.

### FP and SM inhibited SP-mediated upregulation of TNF, CXCL8 and CCL2

**Figure 3:**
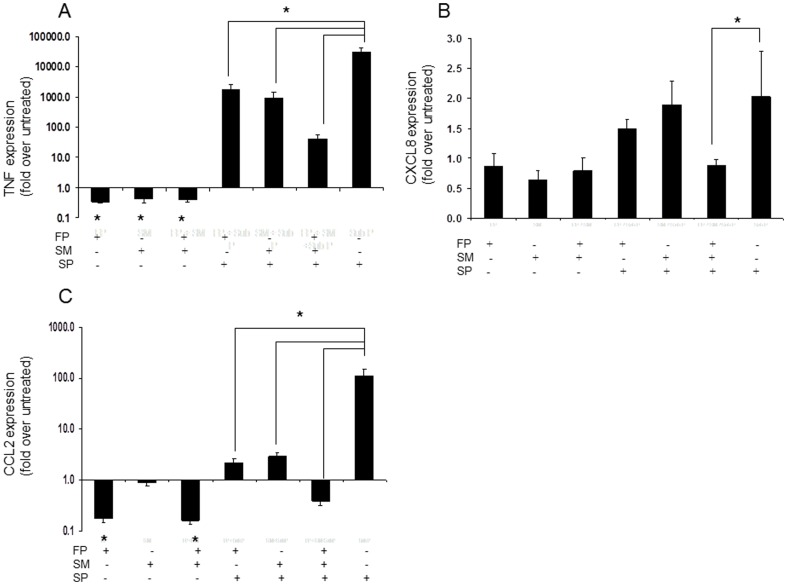
Fluticasone and salmeterol inhibit SP-induced production of TNF and chemokines. **A**, LAD2 human mast cells were treated with fluticasone (1 uM) or salmeterol (1 uM) or combination for 20 hr, then stimulated with SP (1 ug/mL) for 3 hr and TNF, CXCL8 and CCL2 (**A**, **B** and **C** respectively) expression was measured by quantitative RT-PCR (n = 6, * = p<0.01 compared to untreated control, unless otherwise indicated).

Our previous studies have shown that SP activates the expression of TNF, CXCL8 and CCL2 by human mast cells [25]. In the present study, quantitative RT-PCR confirmed SP upregulation of these genes in LAD2 (Fig. 3). Interestingly, FP and SM alone and in combination inhibited constitutive expression of TNF by approximately 50 percent (Fig. 3A). FP and SM alone and in combination also inhibited SP-induced expression of TNF (Fig. 3A). However, FP and SM in combination synergistically inhibited TNF expression, in that their inhibitory effect was about 3 times greater than their additive effect. FP and SM alone and in combination did not inhibit constitutive expression of IL-8/CXCL8 (Fig. 3B). Neither FP nor SM alone had an effect on SP-induced expression of CXCL8. However, SM and FP in combination blocked SP-induced CXCL8 expression completely, reducing CXCL8 levels back to baseline. FP and SM individually inhibited SP-induced expression of CCL2 and in combination their inhibitory effect completely reduced stimulus-induced CCL2 expression below that of the untreated control (Fig. 3C). FP reduced constitutive expression of CCL2 by approximately 70% whereas SM alone had no effect.

### FP and SM inhibited C3a-mediated upregulation of MCP-1/CCL2, RANTES/CCL5 and MIP-1β/CCL4

**Figure 4:**
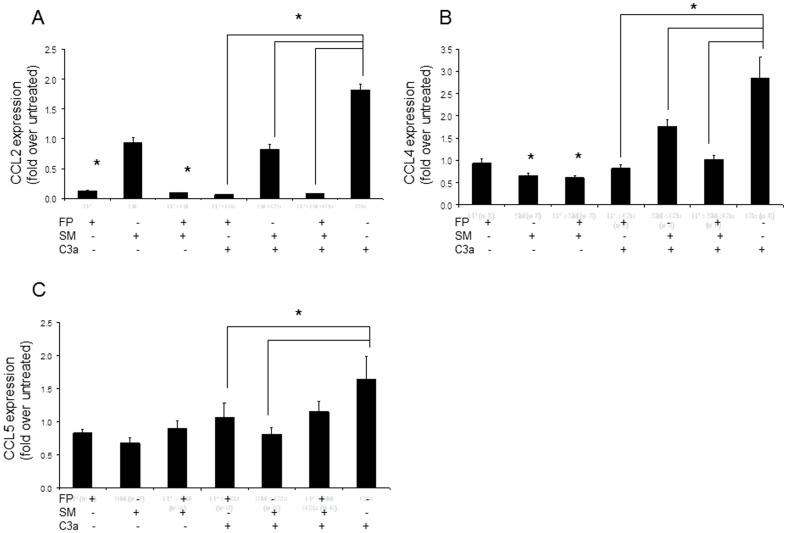
Fluticasone and salmeterol inhibit C3a-induced production of chemokines. **A**, LAD2 human mast cells were treated with fluticasone (1 uM) or salmeterol (1 uM) or in combination for 20 hr and stimulated with C3a (100 ng/mL) for 3 hr and CCL2, MIP-1β/CCL4 and CCL5 (**A**, **B** and **C** respectively) expression was measured by quantitative RT-PCR (n = 6, * = p<0.01 compared to untreated control, unless otherwise indicated).

A preliminary qRT-PCR screen of several genes including TNF, CXCL8, CCL2, CCL5, I-309/CCL1 and CCL4 showed that C3a modestly but significantly upregulated the expression of mRNA for CCL2, CCL5 and CCL4 by LAD2 (data not shown and Fig. 4). C3a upregulated the expression of CCL2 by 2 fold and was not as potent at inducing CCL2 expression as SP, which upregulated CCL2 expression 114 fold (compare Fig. 4A and Fig. 3C). FP and SM alone and in combination inhibited C3a-induced expression of mRNA for CCL2 (Fig. 4A) although the majority of the effect was attributable to FP. FP also inhibited constitutive expression of CCL2 by approximately 90 percent. FP and SM individually and in combination inhibited constitutive expression of CCL4 by approximately 20% (Fig. 4B). Furthermore, FP and SM alone and in combination significantly inhibited C3a-induced CCL4 expression. FP and SM also significantly inhibited expression of CCL5 (Fig. 4C), although this effect was slight. In combination, SM and FP slightly inhibited C3a-induced CCL5 expression although this was not statistically significant. Although the fold changes in mRNA expression of these chemokines and cytokines induced by C3a were modest, these findings were supported at the protein level (see below).

### FP and SM inhibited IgE/anti-IgE-mediated upregulation of CCL2 and CCL1

**Figure 5:**
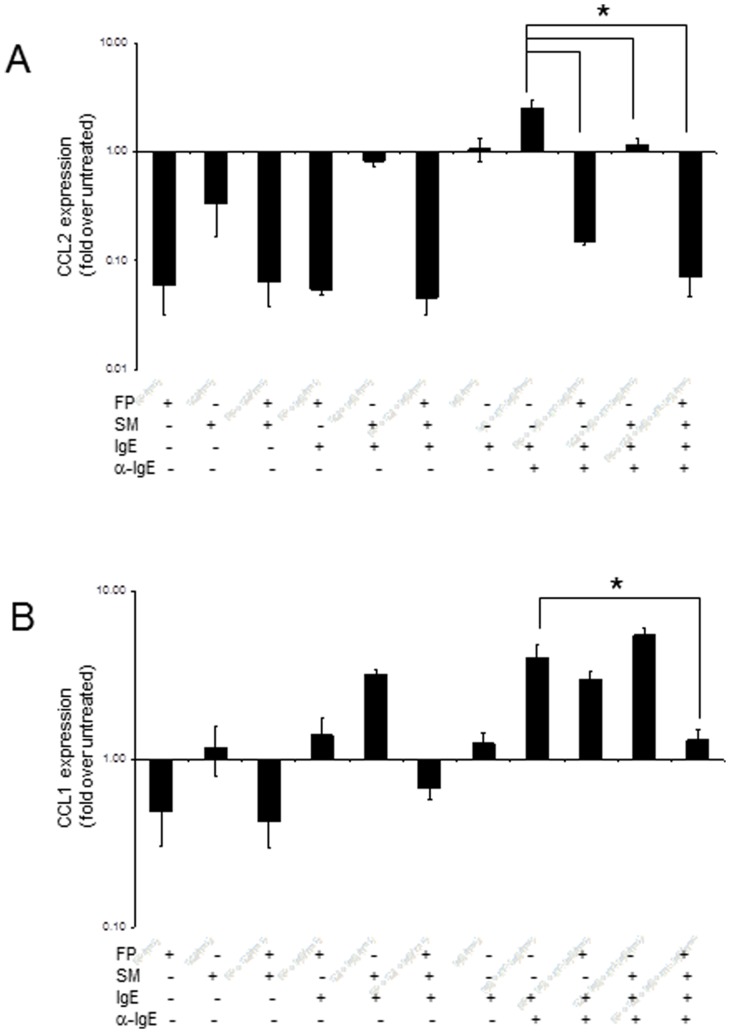
Fluticasone and salmeterol inhibit FcεRI-mediated production of chemokines. **A**, LAD2 human mast cells were sensitized with human myeloma IgE (0.5 ug/mL) for 16 hr, then treated with fluticasone (1 uM) or salmeterol (1 uM) or in combination for 20 hr and stimulated with anti-IgE (100 ug/mL) for 3 hr and CCL2 and CCL1 (**A** and **B** respectively) expression was measured by quantitative PCR (n = 6, * = p<0.01 compared to untreated control, unless otherwise indicated).

Our preliminary qRT-PCR screen further revealed that IgE/anti-IgE stimulation of LAD2 activated expression of CCL2 and CCL1 (data not shown). IgE/anti-IgE upregulated expression of CCL2 mRNA approximately 2 fold (Fig. 5A). Both FP and SM, individually and in combination, inhibited IgE/anti-IgE-induced expression of CCL2. IgE/anti-IgE stimulation also upregulated CCL1 mRNA by approximately 4 fold. FP and SM in combination significantly inhibited IgE/anti-IgE –induced CCL1 expression, whereas neither drug had any effect on their own. FP downregulated constitutive expression of CCL1 whereas SM alone had no effect on CCL1 expression. In fact, the inhibitory effect of the FP and SM combination is likely due solely to FP – on constitutive expression and IgE/anti-IgE-induced expression of CCL.

### FP and SM synergistically inhibited SP-mediated production of TNF and chemokines

**Figure 6:**
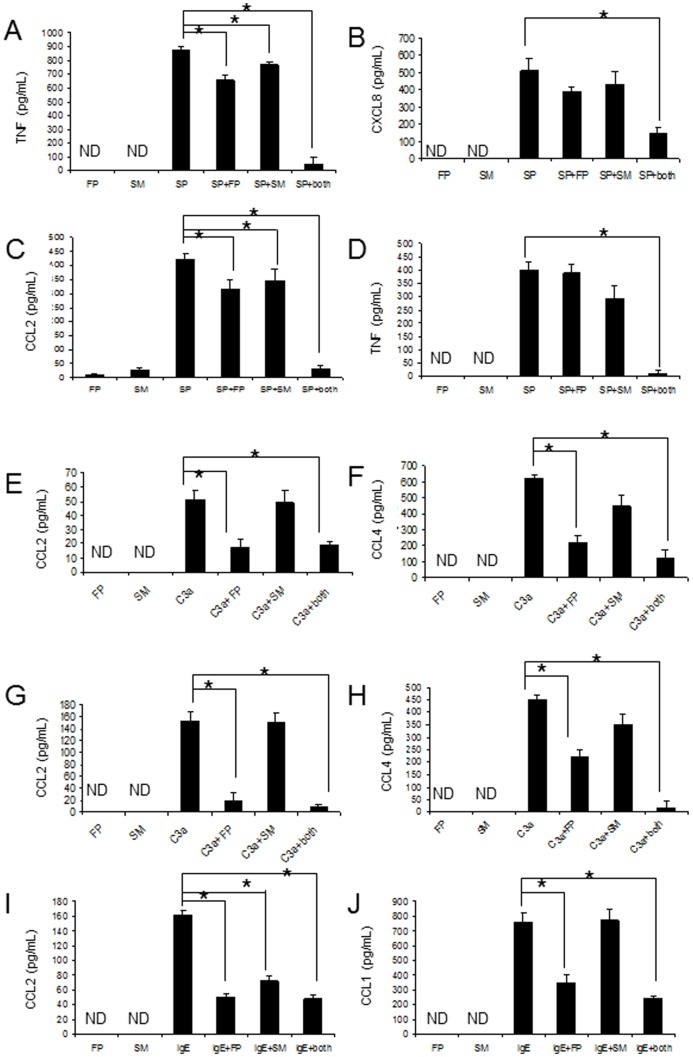
Fluticasone and salmeterol synergistically inhibit SP-mediated production of TNF and chemokines. LAD2 mast cells were treated with fluticasone (1 uM) or salmeterol (1 uM) or in combination for 20 hr and stimulated with SP (1 ug/mL) for 24 hr and TNF, CXCL8 and CCL2 (**A**, **B** and **C** respectively) production was measured by ELISA (n = 3, * = p<0.01). **D**, CD34^+^-derived human mast cells were treated with fluticasone (1 uM) or salmeterol (1 uM) or in combination for 20 hr and stimulated with SP (1 ug/mL) for 24 hr and TNF production was measured by ELISA. (**E** and **F**) LAD2 mast cells were treated with fluticasone (1 uM) or salmeterol (1 uM) or combination for 20 hr and stimulated with C3a (100 ng/mL) for 24 hr and CCL2 and MIP-1β/CCL4 production was measured by ELISA. (**G** and **H**) CD34^+^-derived human mast cells were treated with fluticasone (1 uM) or salmeterol (1 uM) or in combination for 20 hr and stimulated with c3a (100 ng/mL) for 24 hr and CCL2 and MIP-1β/CCL4 production was measured by ELISA. (**I** and **J**) LAD2 mast cells were treated with fluticasone (1 uM) or salmeterol (1 uM) or in combination for 20 hr and stimulated with IgE/anti IgE (1 ug/mL/100 ug/ml) for 24 hr and CCL2 and CCL1 production was measured by ELISA (n = 3, * = p<0.01).

To determine whether FP and SM modified SP-induced production of TNF, CXCL8 and CCL2, mast cell production of these mediators was measured by ELISA. Confirming our previous results [25], SP activated LAD2 to produce significant quantities of TNF, CXCL8 and CCL2(Fig. 6A, B and C). FP and SM alone inhibited SP-induced TNF production only by 18 and 9% respectively (Fig. 6A). However, FP and SM together almost completely inhibited SP-induced TNF production. Similarly, FP and SM alone did not affect SP-induced CXCL8 production, but in combination, FP and SM inhibited SP-induced CXCL8 production by approximately 60% (Fig. 6B). FP inhibited SP-induced CCL2 production by approximately 10% but when combined with SM, FP almost completely inhibited CCL2 production (by 92%; Fig. 6C).

Since LAD2 are a human mast cell line, we next determined if these effects were also observable in primary peripheral blood stem cell-derived human mast cells. SP-induced TNF production was not effected when CD34+-derived human mast cells were pretreated with either FP or SM alone (Fig. 6D). However, FP and SM in combination completely blocked SP-induced TNF production demonstrating the synergistic effects of these two compounds.

### FP and SM synergistically inhibited C3a-mediated production of chemokines

To determine whether FP and SM modified C3a-induced production of CCL2, CCL4 and CCL5, mast cell production of these mediators was measured by ELISA. C3a induced significant production of CCL2 (Fig. 6E) and CCL4 (Fig. 6F) by LAD2 cells and by CD34^+^-derived primary mast cells (Fig. 6G and H). We could not detect significant production of CCL5 by C3a-activated LAD2 cells or CD34^+^-derived primary mast cells (data not shown). SM alone did not affect C3a-induced production of CCL2 or CCL4 by LAD2 mast cells, but FP alone and in combination with SM inhibited C3a-induced CCL2 and CCL4 production (Fig. 6E and F). There was no synergism of FP and SM in this inhibitory effect. Similarly, FP alone and in combination with SM inhibited C3a-induced CCL2 and CCL4 production by CD34+-derived primary mast cells. Once again, SM alone had no effect on C3a-induced production of these chemokines (Figure 6G and H).

### FP and SM synergistically inhibited FcεRI-mediated production of chemokines

To determine whether FP and SM modified FcεRI-dependent production of CCL2 and CCL1/CCL1, mast cell production of these mediators was measured by ELISA. IgE/anti-IgE induced significant levels of CCL2 (Fig. 6I) and CCL1 (Fig. 6J) production by LAD2 cells. FP and SM alone and in combination inhibited FcεRI-mediated production of CCL2. Only FP inhibited FcεRI-mediated production of CCL1 (Fig. 6J).

### FP and SM had no effect on either IgE/anti-IgE or SP-induced production of CysLT

**Figure 7:**
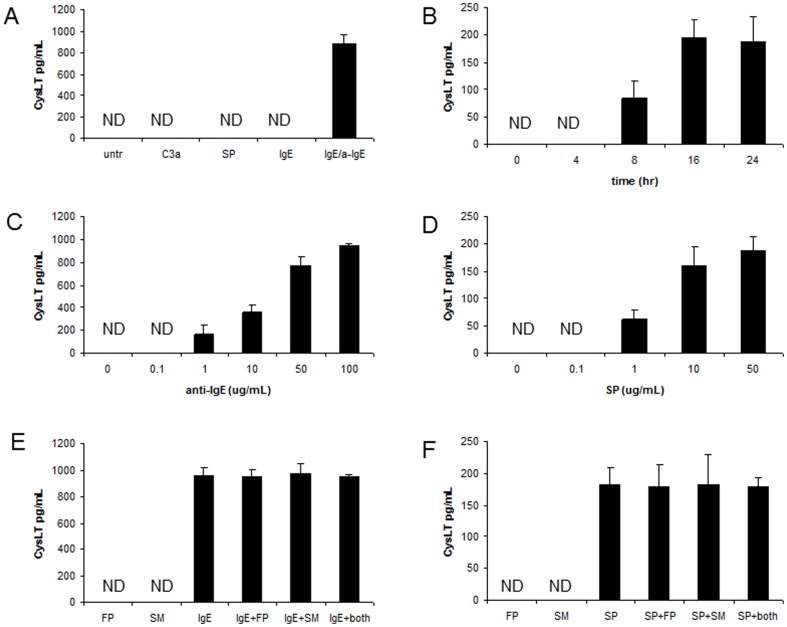
Fluticasone and salmeterol had no effect on FcεRI- and SP-mediated production of cysteinyl leukotrienes. **A**, LAD2 mast cells were stimulated with C3a (100 ng/mL), SP (1 ug/mL), IgE (0.5 ug/mL) or IgE/anti-IgE (100 ug/mL) for 4 hr and CysLT production was measured by ELISA. **B**, LAD2 mast cells were stimulated with SP (1 ug/mL) for the indicated times and CysLT production was measured by ELISA. **C**, LAD2 mast cells were sensitized with IgE (0.5 ug/mL) and stimulated with anti-IgE (100 ug/mL) for 4 hr and CysLT production was measured by ELISA. **D**, LAD2 mast cells were stimulated with SP for 16 hr and CysLT production was measured by ELISA. **E**, LAD2 mast cells were sensitized with IgE (0.5 ug/mL), treated with fluticasone (0.1 uM) or salmeterol (1 uM) for 20 hr, stimulated with anti-IgE (100 ug/mL) for 4 hr and CysLT production was measured by ELISA. **F**, LAD2 mast cells were treated with fluticasone (0.1 uM) or salmeterol (1 uM) for 20 hr and stimulated with SP (1 ug/mL) for 16 hr and CysLT production was measured by ELISA. (n = 3, * = p<0.01).

Cysteinyl leukotrienes (CysLT) production by mast cells is an early and important phenomenon during an acute allergic response. Therefore, we tested the ability of LAD2 to produce CysLT in response to C3a, SP and IgE/anti-IgE stimulation for 4 hr. IgE/anti-IgE induced significant levels of CysLT production, whereas C3a and SP were ineffective (Fig. 7A). CysLT production is dependent upon a series of enzymes such as phospholipase A2 (PLA_2_), 5-lipoxygenase (5-LO) and FLAP. However, it has been shown that SP is able to activate the CysLT production pathway at a level at or beyond PLA_2_, and this effect is mediated, in part, through PKC alpha and beta species [26]. In addition there is evidence to suggest that some SP effects are mediated by 5-LO activation [27], but that induction may possess a slower kinetics to that of IgE/anti-IgE stimulation. Therefore, we stimulated LAD2 cells with C3a and SP for longer than 4 hr and measured CysLT production by ELISA (Fig. 7B). This timecourse analysis revealed that SP induced CysLT production after 8 hr and maximally after 24 hr of treatment. C3a did not induce CysLT production even after 24 hr of incubation (data not shown). An examination of the concentration-dependence of mast-cell activators on CysLT production showed that anti-IgE (100 ug/mL) stimulation produced almost 1 ng of CysLT (Fig. 7C) but SP (10 ug/mL) stimulation produced approximately 10 fold less CysLT (Fig. 7D). FP and SM had no effect on either IgE/anti-IgE or SP-induced production of CysLT (Fig. 7E and F).

### SM downregulated expression of the neurokinin receptors (TACR/NKR)

**Figure 8:**
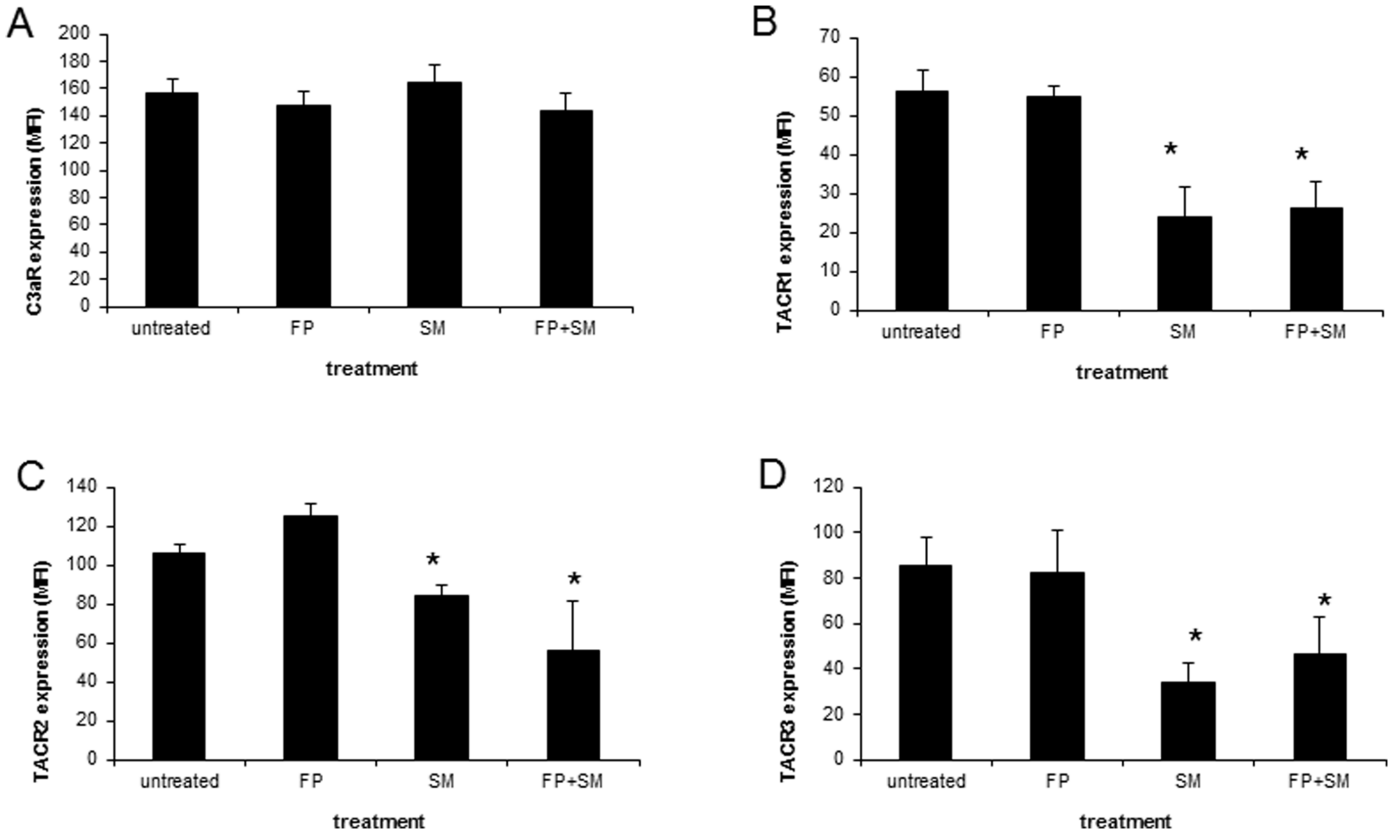
Salmeterol inhibits neurokinin receptor expression. LAD2 human mast cells were treated with fluticasone (1 uM) or salmeterol (1 uM) or combination for 20 hr, and C3aR (**A**), neurokinin receptor 1(TARC1; **B**), 2 (TARC2; **C**) and 3 (TARC3; **D**) expression was measured by flow cytometry. Data are expressed as mean fluorescence intensity (MFI; n = 5, * = p<0.05 compared to untreated control).

Since previous reports have shown that inhaled budesonide downregulates the expression of TACR1/NK-1R in airway smooth muscle cells [28], we examined the expression of C3aR and TACR by LAD2 cells following SM and FP treatment. Neither FP nor SM altered surface expression of C3aR as measured by flow cytometry (Fig. 8A). However, SM downregulated expression of TARC1, TARC2 and TARC3 by approximately 50%.

## Discussion

The mechanisms underlying the beneficial effects of adding long acting β2-adrenoceptor agonists to inhaled corticosteroids in asthma management are not completely understood. In this study, we tested the hypothesis that β2-agonists and corticosteroids synergistically inhibit mast cell mediator release. All three major parameters of mast cell activation were measured including degranulation, cytokine/chemokine production and leukotriene release. The effects of both SM and FP on these parameters were measured in a range of concentrations and time points to determine the precise point at which synergistic effects between these drugs might occur. Initial experiments showed that although FP and SM individually at low concentrations (0.1 nM and 10 nM respectively) had no effect on mast cell degranulation (Fig. 1), in combination they synergistically inhibited SP-induced degranulation. This effect was unique to SP-activated cells since C3a-activated mast cell degranulation was only inhibited by FP, and IgE-mediated degranulation was unaffected by either FP or SM. These results suggest that FP/SM combination treatments would be most apparent in inflammation where SP-producing nerves play a part. This could be the case in chronic asthma in which the immunomodulatory contribution of neuropeptides mediate neurogenic inflammation and neurokinin receptor antagonists decrease airway responsiveness and improve lung function [29].

Our observations are in agreement with previous studies in which both short-acting and long-acting β2-agonists were effective inhibitors of histamine release [30], [31]. However, in contrast to these studies, our data showed that neither FP nor SM were effective at blocking CysLT production (Fig. 7) and it is therefore unlikely that synergistic interactions of these drugs occur in regulation of leukotriene-mediated inflammatory cascades. These results were confirmed in both CD34+-derived human mast cells and the LAD2 human mast cell line. Furthermore, based on the cellular targets of these drugs, we expect that other glucocorticoids and β-agonists would be similarly ineffective at blocking CysLT production. β-agonists inhibit the release of histamine from excised human lung tissue and dispersed human lung mast cells via β2-adrenoceptors; however, this effect is often variable between preparations [32]. The inhibitory effect of β2-agonists is mediated via a sustained increase in cyclic adenosine 5′-monophosphate (cAMP) [33]. Since changes in cAMP affect mast cell degranulation but not membrane proximal events such as PLA2 activation and arachidonic acid metabolism, it is not surprising that SM inhibited mast cell degranulation but had little effect on CysLT production.

Our data further shows that corticosteroids and β-agonists differentially regulate mast cell mediator production. For example, FP was most effective at inhibiting TNF expression, while SM was most effective at inhibiting CCL2 expression. The stimulus used to activate the mast cells also influences the effect of SM and FP on mediator release. For example, FP was very effective at inhibiting production of C3a-induced CCL2 expression, but very poor at inhibiting IgE-mediated CCL1 expression. Furthermore, the synergistic effects of FP and SM were best observed when human mast cells were activated with SP. This suggests that the variable efficacy of FP and SM in patients may be due to heterogeneous mechanisms underlying the inflammatory responses. Patients in whom an inflammatory response is primarily mediated by SP-like neurologic stimuli may be more responsive to FP/SM treatment, while patients in acute stages of an allergic response mediated primarily by IgE and allergen may be less responsive to these drugs.

Among the topical corticosteroids currently available for asthma treatment, fluticasone propionate (FP) has clear efficacy with modest systemic side effects when administered at low concentrations (less than 1000 µg twice a day) [34]. FP exhibits marked anti-inflammatory activity when administered on a regular basis to patients with active asthma, as shown by decreased inflammatory cell infiltrates in bronchial biopsy specimens and bronchoalveolar lavage (BAL) [35]–[37]. For asthma that is inadequately controlled with low to moderate concentrations of inhaled corticosteroids, it is an accepted practice to progressively increase the concentration to achieve disease control. However, because inhaled corticosteroids have a relatively flat concentration-response curve [38], the beneficial effects on inflammation and asthma symptoms might be accompanied by local or systemic side effects. This concern prompted the pioneering studies of Greening *et al*. [39] and Woolcock *et al*. [40], who investigated the effect of adding the long-acting β2-agonist SM (SM) in combination with existing inhaled corticosteroid treatment, compared to the use of higher concentrations of inhaled beclomethasone dipropionate, in asthma patients with persistent symptoms. These and a series of related studies [41]–[43] have clearly demonstrated that the addition of an inhaled long-acting β2-agonist to low or moderate concentrations of inhaled corticosteroids is more efficacious than doubling the concentration of inhaled corticosteroids when assessed by pulmonary function and patient centered-outcome measures.

Lastly, our data confirm numerous previous studies that have shown that glucocorticoids do not inhibit human mast cell degranulation when mast cells are stimulated via FcεRI. For example, dexamethasone in the 10^−6^ and 10^−9^ range has no effect on FcεRI-dependent release of histamine and CysLT from mature cord blood-derived mast cells [12]. Furthermore, dexamethasone treatment does not inhibit the IgE-driven release of mediators (such as histamine and LTC4) by mast cells derived from lung and skin, even at 10^−7^ M [9]. However, mast cells respond differently to glucocorticoids when they are stimulated via receptors other than FcεRI such as G protein-coupled receptors (tachykinin receptors and complement receptors). Prednisolone inhibits SP-activated mouse peritoneal mast cell release of histamine [13] and dexamethasone inhibits mast cell hyperplasia and histamine release [44]. Although a study by Thangam *et al.* showed that C3a-induced mast cell degranulation was not affected by dexamethasone (10 nM), human mast cells were incubated with the glucocorticoid for only 1 hr [45]. In our study, mast cells were pre-treated with fluticasone (Fig. 1D) for 20 hr which allows for sufficient changes in gene transcription, translation and protein production to allow for phenotypic changes.

Our observations are unique in that we have shown that glucocorticoids can inhibit human mast cell degranulation when they are stimulated by non-IgE/FcεRI stimuli such as SP and C3a. Furthermore, we have shown that fluticasone and salmeterol synergistically inhibit SP-activated human mast cell degranulation. Both of these observations are novel and, to our knowledge, have not been previously reported.

The idea that glucocorticoids may influence GPCR-mediated mast cell responses was first suggested two decades ago when it was observed that dexamethasone changed receptor-coupling to G proteins in NECA-mediated responses in RBL-2H3 cells [46]. Exposure of RBL-2H3 cells to dexamethasone attenuated antigen-mediated mast cell degranulation, but potentiated the response elicited by adenosine, mainly by uregulating adenosine receptor A3AR, a GPCR, but also by increasing the expression of G protein alpha i2, alpha i3, alpha s, and beta subunits by two- to three fold [47]. Activation of the A3AR by aminophenylethyladenosine (APNEA) following dexamethasone treatment enhanced the production of inositol phosphates and the mobilization of intracellular Ca^2+^ [47]. Like adenosine receptors, C3aR and tachykinin/neurokinin receptors (TACR1, 2 and 3; receptors for substance P) are G protein-coupled and their signaling is blocked by Pertussis toxin. TACR1/NK1R is upregulated in airway smooth muscle cells in an OVA-challenged rat model of airway inflammation and inhaled budenoside appears to ameliorate this increased expression [28]. We have shown that SM inhibited TACR expression by approximately 50%. Therefore, it is possible that SM inhibits LAD2 responses to substance P by downregulating TACR and this, together with FP effects on immediate signaling events, may synergistically inhibit mast cell activation.

Others have shown that therapeutic concentrations of dexamethasone inhibit intermediate signaling events in RBL-2H3 mast cells, in particular the activation of phosphatidylinositol (PI)3-kinase and downstream signaling events that lead to degranulation [48]. The regulatory p85 subunit of PI3-kinase fails to engage the Grb-associated binder 2 (Grab2) adapter protein which then suppresses the phosphorylation of phospholipase Cγ2 (PLCγ2) and thus reduces calcium flux and degranulation.

The data presented herein demonstrate that FP and SM synergistically inhibit human mast cell degranulation and mediator production (activated by C3a and SP) but not CysLT generation (activated by SP and FcεRI crosslinking). We expect that combinations of other active glucocorticoids and β-agonists will manifest the same positive interaction. These findings reveal the mast cell as an important site of FP and SM interaction and provide further support to the utility of combination therapy in the treatment of mast cell-mediated airways disease.
